# Implementation of a Low-Cost Method to Reduce Bacterial Load in Patient-Room Sink Drains

**DOI:** 10.1017/ash.2021.39

**Published:** 2021-07-29

**Authors:** Emilie Bédard, Marie-Ève Benoit, Thibault Bourdin, Dominique Charron, Gaëlle DeLisle, Stéphane Daraiche, Sophie Gravel, Etienne Robert, Philippe Constant, Eric Déziel, Caroline Quach, Michèle Prévost

## Abstract

**Background:** Sink drains can act as breeding grounds for multidrug-resistant (MDR) bacteria, leading to outbreaks. Drains provide a protected humid environment where nutrient-rich substances are available. Recent and growing installation of water and energy conservation devices have led to increased frequency of drain blockage due to biofilm accumulation. Ineffective drainage may lead to backflow and accumulation of water in the sink during use, increasing the risk of contaminated aerosols formation or direct contamination of surrounding material and equipment. Cleaning and disinfection procedures of sink drains need to be improved to prevent amplification and dispersion of MDR bacteria. The objective of this study was to investigate alternatives to reduce the biofilm and risk of contamination through aerosols. **Methods:** Sink drains from patient rooms were randomly selected in the neonatal intensive care unit of a 450-bed pediatric hospital. We tested 4 approaches: (1) new drain; (2) self-disinfecting heating-vibration drain; (3) chemical disinfection with 20 ppm chlorine for 30 minutes; and (4) thermal disinfection with > 90°C water for 30 minutes. A special device was used during disinfection to increase the disinfectant contact time with the biofilm. Treatments were conducted weekly, with prior sampling of drain water. Other drains were also sampled weekly, including a control drain with no intervention. Bacterial loads were evaluated using flow cytometry and heterotrophic plate counts. The drains were made of stainless steel, a heat-conductive material. **Results:** Preliminary results show that chlorine disinfection had a small impact (<1 log) on culturable bacteria at 48 hours after disinfection but not after a week or repeated weekly disinfection. Thermal disinfection using boiling water is promising, showing an important decrease of 4 log in culturable cells after 48 hours and a concentration still 100× lower 1 week after the disinfection. Repeated weekly thermal disinfection maintained lower culturable levels in the drain. No culturable cells were detected in water from the self-disinfecting drain 2 months after installation, whereas the new drain became fully colonized to concentrations similar to those of drains prior to interventions during the same period. **Conclusions:** Thermal disinfection of drains is a promising alternative to chlorine. This solution is interesting because it is nontoxic and easy to perform, requiring a small volume of hot water. The rapid recolonization of the new drain suggests that replacing contaminated drains is not a sustainable solution and would need to be paired with a thermal disinfection program to maintain low culturable cells.

**Funding:** No

**Disclosures:** None

